# Anesthetic Considerations for Transcarotid Artery Revascularization: Experience and Review of Forty Cases From a Single Medical Center

**DOI:** 10.7759/cureus.12250

**Published:** 2020-12-24

**Authors:** Abistanand Ankam, Sudhakar Kinthala, Praneeth Madabhushi

**Affiliations:** 1 Anesthesia, Guthrie Robert Packer Hospital, Sayre, USA; 2 Anesthesiology, Guthrie Robert Packer Hospital, Sayre, USA

**Keywords:** transcarotid artery revascularization (tcar), anesthetic management of tcar, treatment of carotid stenosis, cerebral oximetry for tcar, hemodynamic management for tcar, review of anesthesia for tcar

## Abstract

Transcarotid artery revascularization (TCAR) procedure is a novel hybrid surgical modality in treating carotid stenosis. Understanding the various steps of the TCAR and the unique challenges involved in the anesthetic management is essential for the successful conduct of anesthesia. In this article, we discuss the overview of the key issues relevant to the anesthetic management and strategies from our experience. We present the data on anesthetic management and outcomes of 40 patients who underwent TCAR procedure at our institute between June 2018 and February 2020. Electronic medical records were retrospectively reviewed and relevant demographic, clinical, and laboratory data were collected. All our patients had general anesthesia with an endotracheal tube utilizing standard American Society of Anesthesiology (ASA) monitoring along with intra-arterial blood pressure monitoring and cerebral oximetry.

The mean age of our patients was 73.6 ± 7.58 years. Fifteen (37.5 %) patients had significant co-morbidities, thus classified as ASA 4 and 10 (25%) patients were on at least three antihypertensives (beta-blockers, calcium channel blockers, angiotensin-converting enzyme inhibitors, loop diuretics, thiazides). Thirty-four (85%) patients were considered to have symptomatic carotid stenosis which was the predominant indication for the TCAR procedure. Patients who had episodes of transient ischemic attack (TIA) or a cerebrovascular accident (CVA) documented by a computerized tomography (CT) scan of the brain and/or residual weakness are considered symptomatic. Thirty-six (90%) of our patients received a bolus dose of 0.2 - 0.4 mg of glycopyrrolate for maintaining heart rate of around 70 beats per minute (BPM) and 38 (95%) received phenylephrine infusion during the carotid clamp to maintain blood pressure between 140 and 160 mm Hg systolic or at patients’ baseline. Twenty-one (52.5%) patients needed antihypertensives such as hydralazine ( 10-20 mg) or beta-blockers such as labetalol (10-20 mg) at the time of emergence from anesthesia to mitigate hemodynamic response during extubation. The mean blood loss was 74 ml ± 33.19 ml, and none of our patients received blood transfusion during the perioperative period. The mean duration of anesthesia was 202.6 ± 27.85 minutes, and the mean length of hospital stay was 1.5 ± 0.97 days.

A thorough preoperative examination with specific attention to the preoperative neurological deficits and cardiopulmonary reserve is important for the meticulous management of intraoperative hemodynamics. Intraoperative administration of glycopyrrolate and the use of vasopressors to maintain optimal hemodynamics to ensure cerebral perfusion during the perioperative period should be considered. The anesthetic goals of carotid revascularization (TCAR) are perioperative hemodynamic stability and early evaluation of neurological status in the immediate postoperative period.

## Introduction

Stroke is the fifth leading cause of death with 140,000 fatalities per year in the United States [1]. Patients with atherosclerotic carotid artery disease are at a higher risk of recurrent ischemic stroke [2]. In a recent study, approximately 41,000 strokes annually were attributed to extracranial internal carotid artery stenosis in the United States [3]. Open carotid endarterectomy (CEA) is the gold standard for the treatment of symptomatic patients with moderate to high-grade carotid artery stenosis (>70%) [4].

Transfemoral carotid stenting (TF-CAS) was introduced as an alternative to the open CEA in patients considered as high-risk due to multiple comorbidities. However, this has become less popular, owing to a higher periprocedural stroke rate compared to CEA [5]. The transcarotid artery revascularization (TCAR) was introduced as an alternative to TF-CAS to eliminate the risk of aortic arch manipulation by directly accessing the carotid artery proximal to the bifurcation. Reversal of arterial flow from the carotid to the femoral vein is then employed for neuroprotection and to minimize the risk of stroke.

The literature for anesthetic management of TCAR is limited. To navigate the unique challenges involved in TCAR, and to achieve favorable outcomes, meticulous anesthetic management is vital. In this article, we discuss the overview of the key issues relevant to the anesthetic management and share our experience and strategies.

## Materials and methods

This is a single medical center retrospective analysis of patient charts from electronic medical records (EMR) for TCAR cases (n=40) between June 2018 and February 2020. The Institutional Review Board (IRB) approval (RB#2001-06) and waiver of informed consent were obtained. EMR was reviewed to obtain information from the anesthesia record, surgical notes, nursing notes, and radiology reports.

The TCAR procedure is a hybrid surgical and endovascular intervention for patients considered high risk for surgical CEA. Patient selection was based on the centers for Medicare and Medicaid services high-risk surgical status [6].

During the preoperative visit, a comprehensive preoperative anesthetic assessment and workup were performed to evaluate the patient’s functional status and identify the severity of comorbidities to optimize them for surgery. Relevant medical and surgical history, airway assessment, and laboratory data were recorded. Vascular workup typically involved a carotid doppler/computerized tomography (CT) angiogram of the neck to identify the extent of stenosis and contralateral vascular patency. The patient’s use of prescription medications with attention to antihypertensives, antiplatelets, and anticoagulants was reviewed. Most patients were on multiple anti-hypertensives and the timing of the last dose of the medication prior to surgery was noted. Typically, medications such as statins and anti-platelets are continued prior to the surgery.

Adequate intravenous access and an arterial line were obtained preoperatively in all the patients. Considering the advanced age of the patient population, most patients did not receive pre-operative benzodiazepines, to minimize drowsiness and enable early neurological examination. On arrival to the operating room, all the patients were connected to standard American Society of Anesthesiology (ASA) monitors along with invasive blood pressure monitoring. Neurological monitoring was performed using cerebral oximetry (Casmed monitor, South Hackensack, NJ, USA) with Fore-sight elite tissue oximetry sensors (Edwards Lifesciences, Irvine, CA, USA). The baseline cerebral oximetry values on room air were noted and it was continuously monitored during the operation. After adequate pre-oxygenation, all the patients were induced with lidocaine (1-2 mg/kg), fentanyl (1-2 mcg/kg), and meticulous titration of propofol (1-2 mg/kg) to loss of consciousness, and succinylcholine (1-1.5 mg/kg) or rocuronium (0.5 -1 mg/kg) was used to achieve neuro-muscular blockade to facilitate laryngoscopy and endotracheal intubation. General anesthesia was maintained with an inhalational agent, along with intermittent boluses of non-depolarizing neuromuscular blocking agents and opioids. The patients were mechanically ventilated and settings adjusted to maintain end-tidal CO_2_ at 30-35 mmHg. A Foley catheter was placed in all patients to monitor urine output. All patients received prophylactic antibiotics prior to surgical incision. Typically, patients received either dexamethasone or ondansetron or both for post-operative nausea and vomiting (PONV) prophylaxis based on the anesthesiologist's preference.

**Figure 1:**
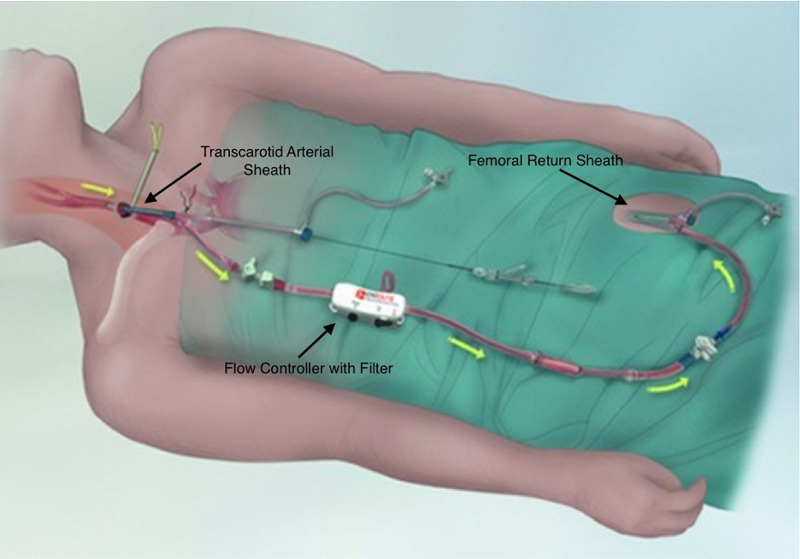
Schematic of the TCAR. Picture reproduced with permission from Silkroad Medical. TCAR: transcarotid artery revascularization.

The procedure involves dissection of the proximal common carotid artery with the insertion of a short sheath, which stays proximal to the carotid bifurcation. As illustrated in Figure 1, the carotid sheath is then connected to a femoral vein sheath through a shunt which has a filter device. To establish the reversal of flow, the common carotid artery is clamped proximal to the insertion of the carotid sheath. Since the femoral vein is at low-pressure compared to the carotid, blood flows in a reverse direction from the brain to the internal carotid artery to the femoral vein through the shunt. During this reversal of flow, any plaque or thrombotic debris is collected at the filter. This eliminates the risk of this debris entering the brain. While the flow is reversed, the lesion is crossed with a wire and treated with angioplasty and stenting [2].

Prior to carotid clamp placement, a phenylephrine infusion to maintain SBP between 140 and 160 mm Hg or at baseline whichever is higher was started. The patients also received glycopyrrolate up to 0.4 mg to maintain a heart rate of around 70 BPM during the cross-clamp and flow reversal period. During cross-clamping, any drop in the cerebral oximetry value of more than 10% was treated with increasing the fractional inspired oxygen (FiO_2_) and augmentation of the blood pressure up to 20% above their baseline. Shortly after releasing the carotid clamp, vasopressors were discontinued in most of our patients as they were not needed. After completion of the procedure, sugammadex (2-4 mg/kg) was used to reverse the neuromuscular blockade, and patients were extubated immediately following the surgery in the operating room upon meeting the standard extubation criteria. A small dose of beta-blocker such as 1-20 mg of labetalol or antihypertensive such a 10-20 mg of hydralazine was used at emergence to mitigate the sympathetic response. A brief neurological examination was performed on all the patients after emergence. Once the patients were in the recovery room, neurological status was monitored according to the post-anesthetic care unit (PACU) protocols.

Data were collected from the anesthetic record for patient demographics, pre-operative chart review, intraoperatively hemodynamic variables, cerebral oximetry data, duration of the carotid clamp, use of anticholinergics, vasoactive agents, and beta-blockers. Other parameters, such as total intravenous fluids, urine output, blood loss, and blood products were also recorded. Further, charts were reviewed to collect information regarding any critical perioperative events or complications. Any relevant complications up to four weeks following the discharge were also noted.

Statistical analysis was conducted for data from the 40 cases (IBM SPSS Statistics for Windows, Version 24.0, IBM Corp., Armonk, NY, USA). Descriptive statistics were obtained for all variables from the data collected. Mean ± SD (standard deviation)) and median (range) were obtained for numerical variables. The number of observations and percentages were obtained for categorical variables.

## Results

We analyzed the data for the 40 patients who underwent TCAR at our facility. Patient demographics, ASA physical status, and preoperative characteristics are shown in Table 1. The mean age of our patients was 73.6 ± 7.58 years. Fifteen (37.5 %) patients were classified as ASA 4 physical status and 10 (25%) patients were on at least three antihypertensives and not all were in the group classified as ASA 4. Thirty-four (85%) patients were considered to have symptomatic carotid stenosis which was the predominant indication for the TCAR procedure. Patients who had episodes of transient ischemic attack (TIA) or a cerebrovascular accident (CVA) documented by a CT scan of the brain and/or residual weakness are considered symptomatic.

**Table 1:** Patient demographics and preoperative data n = designates the number of patients. Values are presented as percentages (%). *Includes patients who had TIA and CVA (includes patients with the residual neurological deficit). Abbreviations: ASA: American Society of Anesthesiology, CAD: coronary artery disease, COPD: chronic obstructive pulmonary disease; OSA: obstructive sleep apnea; GERD: gastroesophageal reflux disease, TIA: transient ischemic attack; CVA: cerebrovascular accident, CEA: carotid endarterectomy, CABG: coronary artery bypass graft, ACE: angiotensin-converting enzyme.

Patient characteristics	Number of patients (n(%))
Age	
<75 years	21 (52.5)
>75 years	19 (47.5)
Sex	
Male	32 (80.0)
Female	8 (20.0)
ASA	
2	1 (2.5)
3	24 (60.0)
4	15 (37.5)
Laterality	
Right	24 (60.0)
Left	16 (40.0)
Preoperative residual neurological deficit	
Yes	15 (37.5)
No	25 (62.5)
Symptomatic*	
Yes	34 (85.0)
No	6 (15.0)
Hypertension	
Yes	34 (85.0)
No	6 (15.0)
CAD	
Yes	29 (72.5)
No	11 (27.5)
Ejection fraction	
<40%	8 (20.0)
>40%	32 (80.0)
Pacemaker	
Yes	5 (12.5)
No	35 (87.5)
COPD	
Yes	5 (12.5)
No	35 (87.5)
Smoking	
Yes	8 (20.0)
No	32 (80.0)
Diabetes	
Yes	18 (45.0)
No	22 (55.0)
OSA	
Yes	5 (12.5)
No	35 (87.5)
GERD	
Yes	15 (37.5)
No	25 (62.5)
Previous CEA	
Yes	9 (22.5)
No	31 (77.5)
CABG	
Yes	6 (15.0)
No	34 (85.0)
Aspirin	
Yes	35 (85.0)
No	5 (15.0)
Plavix	
Yes	34 (85.0)
No	6 (15.0)
Coumadin	
Yes	3 (7.5)
No	37 (92.5)
Other anticoagulants (Eliquis, Brilinta)	
Yes	8 (20.0)
No	32 (80.0)
Beta-blockers	
Yes	27 (67.5)
No	13 (32.5)
Calcium channel blockers	
Yes	12 (30.0)
No	28 (70.0)
ACE inhibitors	
Yes	24 (60.0)
No	16 (40.0)
Diuretics	
Yes	6 (15.0)
No	34 (85.0)
Thiazides	
Yes	5 (12.5)
No	35 (87.5)
Statins	
Yes	36 (90.0)
No	4 (10.0)

Only three (7.5%) of our patients received preoperative benzodiazepines. All our patients were managed under general anesthesia with endotracheal intubation. Data for anesthetic time, clamp time, intraoperative fluids, blood loss, and urine output are shown in Table 2. Thirty-six (90%) patients received glycopyrrolate intraoperatively. Prior to clamp placement, all our patients (100%) were started on phenylephrine infusion for maintenance of SBP between 140 and 160 mm Hg. Thirty-eight (95%) received phenylephrine during the carotid clamp. However, only four (10%) patients required continuation of the phenylephrine infusion after releasing the clamp. Twenty-two (55%) patients needed the use of either a beta-blocker or hydralazine during emergence to mitigate hemodynamic response. The mean estimated blood loss was 73 ± 33.19 ml, and none of the patients required transfusion of blood products intraoperatively.

**Table 2:** Intraoperative parameters: anesthetic time, clamp time, intraoperative fluids, blood loss, and urine output. Values are presented as mean (SD). Abbreviation: SD = standard deviation.

Intraoperative parameters	Mean ± SD
Anesthesia time (minutes)	202.6 ± 27.85
Clamp time (minutes)	16.6 ± 9.02
Fluids (ml)	2226.3 ± 467.81
Blood loss (ml)	73.6 ± 33.19
Urine output (ml)	513 ± 228.08

All the patients were extubated in the operating room. The average length of hospital stay was 1.50 ± 0.97 days. Twenty-eight (70 %) patients were discharged on postoperative day (POD) 1, another seven patients (17.5%) were discharged on POD 2, and only five patients stayed in the hospital for three or more days. One of our patients who presented with an evolving stroke underwent TCAR, had a hospital stay of five days.

Postoperative complications (PACU/ICU) are shown in Table 3. Following hospital discharge, we noted complications of hoarseness of voice, speech difficulty, hypertensive emergency, and myocardial ischemia during our chart review which were reported to surgical quality. None of our patients had a stroke or death. Follow up was done at one week and four weeks post-procedure.

**Table 3:** Postoperative complications/events, management, and outcome. n = designates the number of patients. Abbreviations: PACU: postanesthesia care unit, ICU: intensive care unit.

Complication	Location	Number (n)	Management	Outcome
Hypotension	PACU	4	Fluids bolus and phenylephrine infusion	Resolved <12 hours
Hypertension	PACU	5	Nicardipine infusion and administration of beta-blocker	Resolved in <12 hours
Chest pain	PACU	1	Cardiac workup negative - conservative management	Resolved in < 2 hours
Speech difficulty	PACU	1	Neuro workup negative - conservative management	Resolved in 24 hours
Neck swelling	PACU	1	Coagulation and ACT normal pressure bandage applied	Resolved in < 2 hours
Groin hematoma	PACU	1	Hematoma progressed to retroperitoneal space - conservative management	Discharged after 2 days
Bradycardia	ICU	3	B-blockers withheld	B-blocker dose reduced
Cough	ICU	1	Conservative management	Spontaneous resolution <24 hours
Hoarseness of voice	ICU	1	Conservative management	Spontaneous resolution <24 hours

## Discussion

Literature is sparse regarding the anesthetic management of TCAR. Our objective is to share our experience and discuss the key anesthetic events in the management of TCAR for optimal outcomes.

Patients may present to the operating room within two weeks of a CVA/TIA as the evidence is strong in decreasing recurrent strokes [2]. Baseline assessment of pre-operative neurological deficit is important to identify any new deficits post-procedure. A significant proportion of patients have underlying cardiac dysfunction; thus, specific attention to left ventricular ejection fraction (LVEF) is vital to alert providers about the potential low-flow circulation and delayed onset of action of medications, thus needing careful titration.

The TCAR procedure can be performed either under general anesthesia, regional anesthesia, or monitored anesthesia care (MAC). General anesthesia with the endotracheal tube will have the advantage of providing optimal surgical conditions with patient positioning, immobility, and secured airway with controlled ventilation and oxygenation. The advantages of performing TCAR under regional or MAC include reliable neurological monitoring, decreased sympathetic stimulation associated with airway manipulation, and minimizing cough during extubation, which may potentially disrupt the sutures and cause neck hematoma.

A retrospective review by Button et al. reported 73.4% of the cases being done under MAC. However, it is noted that the use of MAC decreased from 80% in 2012 to only 40% by 2016, showing a trend toward increasing use of general anesthesia [7]. The mean hospital stay was around 2.99 days with MAC versus 4.3 days with general anesthesia. Also, other recent studies including the carotid revascularization endarterectomy versus stenting trial (CREST) showed a higher incidence of perioperative cardiac complications in the general anesthesia group versus the local [8-10]. However, the general anesthesia versus local anesthesia for carotid surgery (GALA) trial which looked at the outcomes of carotid surgery following general anesthesia versus local anesthesia found no significant differences in stroke, death, and myocardial infarction (MI) between the groups [11].

To perform TCAR under local anesthesia and sedation, the patients must be cooperative and remain still during the procedure. Hypoventilation can lead to CO_2_ retention and can potentially worsen pulmonary pressures leading to significant cardiovascular instability. Therefore, it may be challenging to provide moderate sedation in patients with COPD, obstructive sleep apnea (OSA), and elevated pulmonary pressures. Most cases in hybrid rooms with fluoroscopy where access to the patient is challenging. If airway intervention is needed, the imaging arm of the fluoroscope positioned over the face of the patient may limit access, not to forget that the surgical site is the neck. Considering the aforementioned reasons all our cases were performed under general anesthesia.

Regardless of the anesthetic technique, the goals of carotid revascularization (TCAR) are hemodynamic stability and the ability to evaluate neurological status in the immediate postoperative period. A large prospective randomized control trial is needed to delineate the difference in outcomes secondary to the type of anesthesia.

Carotid sinus manipulation is inevitable during the TCAR procedure. Wide swings in blood pressure in the perioperative period are frequent during carotid surgery because of the plaque being commonly located in the area of the carotid sinus [12,13]. Manipulation of the carotid sinus during open carotid endarterectomy can lead to significant hemodynamic instability, often accompanied by sudden bradycardia [10]. During an open carotid endarterectomy, with surgical disruption of the nerve endings, the resultant hemodynamic instability which can occur in up to 55% of the patients, may last up to hours to days [13].

During carotid artery stenting, secondary to balloon angioplasty, and stent placement, the resultant stimulation of the carotid body receptors can lead to hypotension and bradycardia in 76% of the patients [13]. This could last up to 12 to 24 hours. These patients present a higher risk for cardiovascular and cerebrovascular complications [13]. The intraprocedural hemodynamic instability is usually transient and self-limiting. Administration of prophylactic anticholinergics would help in mitigating bradycardia associated with carotid sinus manipulation. We administered glycopyrrolate for our patients undergoing TCAR, as it has a longer half-life and better cardiovascular adverse effect profile [14].

The gold standard for neuromonitoring is performing an intraoperative neurological examination. This is possible only when the procedure is performed under local anesthesia, and the patient is cooperative. Neuromonitoring under general anesthesia can be done in several ways, including electroencephalogram (EEG) monitoring, cerebral oximetry, transcranial Doppler (TCD), and somatosensory evoked potentials (SSEP) [4].

Cerebral oximetry is based on near-infrared spectroscopy, which measures hemoglobin oxygen saturation in mixed arterial, capillary, and venous blood in the frontal tissue bed illuminated by near-infrared light [15]. The reduction in cerebral oximetry was shown to correlate with changes in EEG, TCD, SSEP, and postoperative neurological deficits [15]. Presently, there is no consensus on the cut-off value for predicting cerebral ischemia. The reduction in cerebral oximetry values more than 12% from a baseline preoperative value has been identified as a reliable, sensitive, and specific threshold for the detection of brain ischemia [16]. By maintaining the SBP between 140 and 160 mm Hg or 20% above the baseline whichever is higher, the cerebral oximetry readings returned to the pre-clamp values with an improved collateral circulation across the circle of Willis.

Carotid reversal of flow is the critical phase of the procedure. It is of utmost importance to maintain the SBP between 140 and 160 mmHg and heart rate about 70 BPM to maintain adequate cardiac output. Any drop in the cerebral oximetry value of more than 10%, can be treated with either increasing the FIO_2_ or augmentation of blood pressure up to 20% above their baseline or both.

Early neurological assessment after extubation and during the immediate postop period is vital. Attention needs to be given to hemodynamics in the post-operative period as persistent stimulation from the carotid stent can lead to sustained hypotension and bradycardia. Postoperatively, hyper- and hypotension should be avoided to prevent hyperperfusion syndrome or stent thrombosis respectively [17]. Monitoring of the neck and groin access site for hematoma is important. Specific attention to bleeding complications is integral in the perioperative period since most patients are on dual antiplatelet therapy and are therapeutically heparinized intra-operatively.

Postoperative complications of TCAR include bleeding, infection, cranial nerve injuries, myocardial ischemia/ infarction, CVA, and death. Literature showed a stroke rate of TCAR from the safety and efficacy study for Reverse Flow Used During Carotid Artery Stenting Procedure (ROADSTER) trial is 1.4%, which is the lowest of any prospective clinical trial of carotid stenting [3]. One possible benefit of TCAR is a lower incidence of cranial nerve injuries compared to CEA (0.3% vs. 3.8%; p = 0.01) [18].

The present study has several limitations. As this is a single-center retrospective analysis of data, any errors with the documentation and retrieval of data from charts could influence the results and information. Our sample size is small to draw any major conclusions or to make any recommendations. Prospective randomized trials with large sample sizes are needed to draw any strong recommendations.

## Conclusions

A thorough preoperative examination with specific attention to the preoperative neurological deficits and the cardiopulmonary reserve is important for the meticulous management of intraoperative hemodynamics. Intraoperative administration of glycopyrrolate and the use of vasopressors to maintain optimal hemodynamics to ensure cerebral perfusion during the perioperative period should be considered. The anesthetic goals of carotid revascularization (TCAR) are perioperative hemodynamic stability and early evaluation of neurological status in the immediate postoperative period.
